# Climate change perception and pro-environmental behavior among urban park visitors: the mediating role of place attachment

**DOI:** 10.3389/fpsyg.2025.1685855

**Published:** 2025-12-11

**Authors:** Jee In Yoon, Jinyoung Joo, Soyoun Lim, Eun Seo Kim, Chang Mi Lee

**Affiliations:** 1 Department of Coaching, College of Physical Education, Kyung Hee University, Yongin-si, Republic of Korea; 2 Center for Happiness Studies, Seoul National University, Seoul, Republic of Korea; 3 Department of Kinesiology, Mississippi State University, Starkville, MS, United States

**Keywords:** climate change, place attachment, nature-based recreation, park, pro-environmental behavior

## Abstract

**Objectives:**

This study investigates how climate change perception influences pro-environmental behavior—on-site (park-specific) and off-site (daily life)—among urban park visitors, focusing on the mediating role of place attachment (place identity, place dependence, and social bonding).

**Methods:**

A survey was conducted with 351 adults engaging in walking and running at Namsan Park in Seoul. Measures included climate change perception, place attachment, and pro-environmental behaviors. Structural equation modeling and bootstrapping were used for analysis.

**Results:**

Climate change perception significantly predicted all three dimensions of place attachment. Place identity and social bonding mediated the relationship between climate change perception and both types of pro-environmental behavior. Place dependence was significantly associated with on-site, but not off-site, pro-environmental behavior.

**Conclusion:**

Place attachment serves as a key mechanism linking climate concern to sustainable actions in leisure settings. Strengthening emotional and social bonds to urban parks may enhance both localized and general pro-environmental behaviors, offering practical implications for urban sustainability strategies.

## 1 Introduction

Despite historically lower levels of climate change risk perception in Asia compared to Europe and North America, recent trends indicate a notable shift in public awareness. For instance, only about 31% of respondents in developing Asian countries viewed climate change as a very serious threat in the early 2010s (Lee et al., 2015), but this awareness has grown significantly in more developed contexts such as South Korea. The increasing frequency of extreme weather events and environmental crises has also contributed to greater recognition of climate risks, which has begun to influence not only daily life but also leisure behaviors (Evans, 2019). Recreationists are increasingly selective about the destinations they visit, opting for eco-friendly or less carbon-intensive leisure options. According to a survey by the European Travel Commission (2024), 76% of European travelers have modified their holiday plans due to concerns about climate change, with younger tourists being particularly responsive to extreme weather risks and rising temperatures (Rankin, 2024). Additionally, interest in nature-based activities—such as hiking, cycling, and forest bathing—has grown, alongside stronger support for conservation efforts and sustainable use of park environments (Wilkins and Horne, 2024).

**Figure 1:**
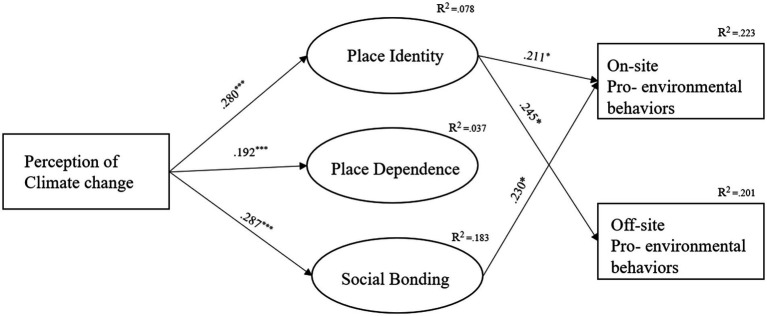
Final model estimation.

Several studies have shown that climate change perception positively predicts pro-environmental behavior (PEB) and leisure-related decisions. For example, Park and Chang (2024) found that South Korean adults who perceive climate change as an imminent threat exhibit greater willingness to reduce consumption and adopt sustainable practices. Similarly, Ziegler (2017) showed that belief in anthropogenic climate change significantly correlates with individual support for climate policy and behavioral action. In the context of leisure, Han et al. (2016) observed that climate concern shapes tourist behaviors, particularly in nature-based tourism. Through these studies, it becomes evident that individuals’ perceptions of climate change significantly influence not only their daily lives but also their leisure activities (Figure 1).

While climate change perception broadly influences PEB, it is important to distinguish between everyday actions and context-specific behaviors that occur in leisure settings. Song and Soopramanien (2019) emphasized that park visitors exhibit both on-site environmentally responsible behaviors (e.g., proper waste disposal) and more general off-site attitudes, with differing predictors. Chan et al. (2017) found similar patterns among users of urban green spaces, suggesting a nuanced understanding of environmental behavior is necessary in park-related research. Yoon et al. (2024) further supported this distinction in their study of Bukhansan National Park visitors in South Korea, demonstrating that while pro-environmental attitudes influenced both behavioral domains, place attachment significantly mediated on-site environmentally responsible behavior more strongly than general actions. Together, these findings underscore the importance of accounting for behavioral context and psychological attachment to place when examining the effects of climate-related attitudes on environmental behavior in leisure settings.

In particular, the growing attention to place attachment in recent studies highlights its potential as a key explanatory mechanism linking environmental concern to actual behavioral outcomes in leisure contexts. Place attachment—conceptualized as place identity, place dependence, and social bonding—is a key mediating factor that connects environmental attitudes with behavior (Scannell and Gifford, 2010). Place identity refers to the emotional and symbolic meanings individuals associate with a place, shaping their sense of self. Place dependence describes the functional value of a place in fulfilling one’s specific goals or activities more effectively than alternative settings. Social bonding captures the interpersonal connections and shared experiences that form in a particular place. Empirical research supports this link in leisure settings. For example, Lee and Shen (2013) demonstrated that place identity and dependence significantly mediate the relationship between environmental concern and conservation behaviors among visitors to Bukhansan National Park in Korea. Additionally, Ramkissoon et al. (2013) showed that social bonding within recreational settings strengthens environmentally responsible behavior. Taken together, these findings illustrate that place attachment is not merely a passive emotional response but a critical psychological mechanism that channels climate-related attitudes into concrete environmental behaviors—especially within leisure and nature-based contexts.

To guide this study, we adopt the Value-Belief-Norm (VBN) theory as the primary theoretical framework. VBN explains how environmental values and beliefs shape moral norms that, in turn, drive PEBs. In this perspective, *climate change perception* can be viewed as a critical belief that activates behavioral commitments. By introducing place attachment as a mediating mechanism, this study extends the VBN framework to the context of urban park leisure, illustrating how environmental concern translates into both on-site and off-site sustainable actions.

Therefore, this study aims to investigate the structural relationship among climate change risk perception, place attachment, and PEB among visitors to Namsan Park, a natural urban leisure space in Seoul. Specifically, the study examines how place identity, dependence, and social bonding mediate the effect of climate change perception on both on-site (park-specific) and off-site (daily life) pro-environmental actions. This research contributes to leisure and environmental psychology literature by elucidating the emotional and functional mechanisms that promote sustainable behavior in urban green environments. Practically, the study offers insights into urban park planners and policy makers aiming to foster environmental stewardship through place-based engagement.

*Hypothesis 1*: Climate change perception will positively influence place attachment (place identity, place dependence, and social bonding).

*Hypothesis 2-1*: Place identity will positively influence on-site PEB.

*Hypothesis 2-2*: Place identity will positively influence off-site PEB.

*Hypothesis 2-3*: Place dependence will positively influence on-site PEB.

*Hypothesis 2-4*: Place dependence will positively influence off-site PEB.

*Hypothesis 2-5*: Social bonding will positively influence on-site PEB.

*Hypothesis 2-6*: Social bonding will positively influence off-site PEB.

*Hypothesis 3*: The relationship between climate change perception and on-site and off-site PEBs will be mediated by place attachment (place identity, place dependence, and social bonding).

*H4-1*: Climate change perception will positively predict on-site PEB.

*H4-2*: Climate change perception will positively predict off-site PEB.

## 2 Literature review

### 2.1 Climate change

Climate change is closely associated with extreme temperatures, air pollution, ecosystem degradation, and the increased frequency of natural disasters. These environmental changes contribute to various acute and chronic health conditions and are recognized as risk factors for premature mortality (Lelieveld et al., 2019; Watts et al., 2019). Thus, climate change has emerged as a major societal issue that directly affects human health and quality of life, extending far beyond environmental concerns. To understand how individuals respond to climate-related phenomena, it is essential to consider the role of perception. Perception refers to the psychological response formed based on an individual’s knowledge and experience regarding a specific event (Anderson, 2004). Accordingly, “climate change perception” can be defined as an individual’s cognitive and emotional appraisal of climate change based on accumulated experiences and understanding (Van der Linden, 2015).

Recent studies have shown that climate change perception functions as a critical psychosocial factor, influencing not only awareness but also mental health (Chae et al., 2024). This perception encompasses more than the passive reception of information—it shapes emotional responses such as climate anxiety and motivates adaptive behaviors. In South Korea, public attention to climate anxiety has been increasing (Son and Lee, 2022; Jang, 2022). This heightened awareness suggests that individuals’ climate change perception influences both cognitive evaluations and affective reactions. Notably, Chae et al. (2024) found that climate anxiety does not merely represent a negative emotional state; instead, it can function as a catalyst for adaptive coping and PEB. Experiencing a certain degree of climate anxiety may be a natural psychological response to the current climate crisis and may serve as a meaningful driver for climate action. Therefore, measuring individuals’ perceptions and emotional responses toward environmental threats is essential for developing effective environmental policies and interventions.

### 2.2 Place attachment

Place attachment refers to the emotional and functional bond individuals develop with specific places through personal experiences, symbolic meanings, and social interactions. It is widely recognized as a significant factor influencing human behavior in settings such as parks, neighborhoods, and recreational spaces (Ramkissoon et al., 2013). The concept originates from Bowlby and Solomon’s (1989) attachment theory, which posits that emotional ties in interpersonal relationships can also extend to physical environments. Building on this foundation, place attachment is typically conceptualized through two main dimensions: place identity and place dependence (Williams and Vaske, 2003). Place identity reflects the symbolic and emotional meanings attributed to a place—how it becomes part of an individual’s values, memories, and sense of self. In contrast, place dependence refers to the functional importance of a place in enabling preferred activities or satisfying specific needs. This functional attachment is closely linked to PEBs that are specific to particular locations, such as proper waste disposal or park conservation efforts (Kyle et al., 2004b). In addition, Kyle et al. (2004a) introduced social bonding as a third subdimension, referring to interpersonal relationships and shared experiences that strengthen emotional ties to place and reinforce environmentally responsible behaviors.

Previous research also indicates that emotional connectedness to nature—such as empathy with nature and nature relatedness—mediates the relationship between place attachment and PEB (Mayer and Frantz, 2004; Qu et al., 2019). Moreover, place attachment serves as a psychological buffer that enhances resilience to climate change and promotes support for environmental policies, particularly when the place is perceived to be under threat. From this perspective, place attachment can act both as a mediator—translating concern for climate issues into behavioral responses—and as a moderator—intensifying the effect of social and environmental norms on PEB (Lee and Shen, 2013; Ramkissoon et al., 2013).

### 2.3 Pro-environmental behavior (PEB)

Pro-environmental behavior (PEB) encompasses actions that contribute positively to the environment, including energy conservation, recycling, reduced consumption, and the use of sustainable products (Turaga et al., 2010). These behaviors are considered vital for mitigating environmental degradation and promoting sustainability, and they have been widely studied across disciplines including psychology, sociology, and economics (Brick et al., 2024). To explain PEB, several theoretical approaches have been proposed. From a social psychological standpoint, internalized personal norms are considered the strongest predictors of PEB, while external social norms exert indirect effects through these internal values (Thøgersen et al., 2023). Rational choice models such as the Theory of Planned Behavior emphasize the roles of attitudes, perceived behavioral control, and self-efficacy in shaping behavior (Turaga et al., 2010). Value- and identity-based approaches further highlight the role of altruistic, biospheric values and environmental identity in motivating environmentally responsible actions (Gatersleben et al., 2014).

Behavioral economics also contributes to understanding PEB by examining preferences such as altruism, reciprocity, and patience influence decision-making (Lades et al., 2021). Additionally, recent research emphasizes the role of self-transcendent emotions—such as awe, gratitude, and nature connectedness—in fostering sustainable behavior (Zelenski and Desrochers, 2021). The concept of behavioral spillover is another important perspective, suggesting that engaging in one type of PEB can lead to the adoption of additional behaviors, particularly when intrinsic motivation and perceived similarity between behaviors are high. However, scholars caution that spillover effects are not always positive and may sometimes reduce engagement in other environmental actions (Behn et al., 2025).

Place attachment has emerged as a meaningful psychological determinant of PEB. Individuals with strong emotional bonds to places are more likely to engage in environmentally responsible behaviors (Larson et al., 2018; Lee, 2011). Stedman (2002) further argued that place-based cognition and identity foster the motivation to protect valued environments. Thus, emotional ties to place serve as both motivational and cognitive foundations for sustained PEB.

### 2.4 Theoretical framework

The present study is grounded in the Value-Belief-Norm (VBN) theory, a widely applied model in environmental psychology that explains environmentally significant behavior through a sequential chain of values, beliefs, and norms (Stern, 2000). According to VBN, environmental actions are motivated when individuals’ value orientations (e.g., altruistic, biospheric) shape their ecological worldviews and beliefs about human–environment relationships, which in turn activate a sense of moral obligation to act. These activated personal norms ultimately predict specific PEBs.

Within this framework, climate change perception represents a belief structure that signals environmental threat and triggers pro-environmental norms. Numerous studies support this mechanism, showing that individuals with stronger climate-related beliefs are more likely to engage in sustainable practices and support conservation policies (Dietz et al., 1998; Steg and de Groot, 2010).

The contribution of this study lies in extending the VBN model by incorporating place attachment as a mediating mechanism. While VBN emphasizes the cognitive–normative pathway, it pays less attention to the emotional and social bonds that connect people to specific environments. Drawing on research that highlights the importance of place identity, place dependence, and social bonding (Scannell and Gifford, 2010; Ramkissoon et al., 2013), we argue that these dimensions of attachment strengthen the link between climate-related beliefs and concrete pro-environmental actions. For example, individuals who perceive climate change as a serious threat may develop stronger emotional and functional ties to parks, which then motivate both localized conservation behaviors (on-site) and broader lifestyle practices (off-site).

By embedding place attachment within the VBN framework, this study contributes to a richer understanding of the psychological processes that channel climate concern into sustainable leisure behaviors in urban settings.

### 2.5 Hypotheses development

Based on the Value-Belief-Norm (VBN) framework and prior empirical research, we developed the following hypotheses.

*H1*: Climate change perception and place attachment.

According to VBN theory, environmental beliefs are precursors to norms and behavior (Stern, 2000). When individuals perceive climate change as a serious risk, they are more likely to form stronger emotional and functional bonds with meaningful places (Scannell and Gifford, 2010). Empirical work in park and tourism contexts also links place-based cognitions/attachments with environmentally responsible orientations (Halpenny, 2010; Ramkissoon et al., 2013; Williams and Vaske, 2003). Therefore, we hypothesize that climate change perception will positively influence all three dimensions of place attachment.

*H2*: Place attachment and PEBs.

Place attachment has consistently been linked to environmentally responsible behavior. Place identity motivates individuals to engage in conservation actions as a way of protecting spaces that form part of the self (Stedman, 2002). Place dependence reflects the functional importance of a setting, which can encourage context-specific actions such as waste reduction or conservation practices in parks (Kyle et al., 2004b). Social bonding emphasizes the interpersonal dimension of attachment, creating social norms that reinforce sustainable behavior (Ramkissoon et al., 2013). Based on these insights, we hypothesize that the three subdimensions of place attachment will each positively predict both on-site and off-site PEBs.

*H3*: Mediation of place attachment.

While the VBN model highlights a cognitive–normative pathway from beliefs to behaviors, subsequent research emphasizes the role of emotional and relational processes in strengthening this link (Scannell and Gifford, 2010). Place attachment functions as such a mediator by translating environmental concern into concrete actions. Thus, we hypothesize that the relationship between climate change perception and PEBs will be mediated by place attachment.

*H4*: Direct effects from climate change perception to PEB.

Classic approaches once required a significant total effect from beliefs to behavior before testing mediation. However, contemporary mediation practice emphasizes the significance of the indirect effect estimated via bootstrapping and does not require a significant total effect (or even a direct effect) as a precondition (MacKinnon, 2008). Still, to clarify the behavioral pathways in our context, we specify and test direct paths from climate change perception to both behavioral domains.

## 3 Methods

### 3.1 Data collection

This study was conducted with visitors to Namsan Park in Seoul, South Korea, focusing specifically on individuals engaging in walking and running activities during their leisure time. Participants were recruited using a non-probability purposive sampling method, targeting adults who were actively walking or running along the park trails. The inclusion criteria required participants to be at least 18 years of age and able to provide informed consent. Data were collected over a 4-day period—two weekdays and two weekend days—in September and October 2023, at various times of day to ensure participant diversity. Three trained research assistants, who had been instructed in the study protocol and research ethics, approached potential participants on-site. After providing a clear explanation of the study’s purpose and obtaining verbal informed consent, the assistants distributed paper-based questionnaires to those who voluntarily agreed to participate. Completion of the survey took approximately 10 to 15 min, and no compensation was offered for participation. A total of 373 questionnaires were collected. After excluding 16 responses due to incomplete or inconsistent answers, a final sample of 351 valid responses was used for data analysis. An *a priori* power analysis was conducted in G*Power 3.1 for SEM planning using a multiple-regression framework (fixed model, *R*^2^ deviation from zero). With ƒ^2^ = 0.15 (medium), *α* = 0.05, power = 0.95, and four predictors, the required minimum sample size was *N* = 129 (Cohen, 2013; Faul et al., 2009). This is consistent with methodological recommendations indicating that SEM models typically require between 200 and 500 participants depending on model complexity (Wolf et al., 2013).

### 3.2 Ethical considerations

This study was approved by the Institutional Review Board of Kyung Hee University (KHGIRB-23-380). All participants were informed about the purpose of the study and provided written informed consent prior to participation, and all research procedures adhered to the ethical guidelines for research involving human participants.

### 3.3 Measures

Climate change perception was measured using four items designed to assess individuals’ awareness and concern regarding environmental changes associated with climate issues (Van Valkengoed et al., 2021). A total of four items captured participants’ perceptions of climate change (e.g., “I feel that air pollution is becoming more serious”). Place attachment was measured using items adapted from previous studies (Kyle et al., 2005; Kyle et al., 2004a) and included dimensions of place identity (three items measuring emotional and symbolic connection to the park, such as “I have a strong sense of belonging in regard to Namsan Park”), place dependence (four items measuring functional attachment to the park, such as “I am more satisfied with visiting Namsan Park than other destinations”), and social bonding (two items measuring social interaction within the park, such as “I have many pleasant memories with acquaintances in Namsan Park”). Lastly, PEB was measured in two subdimensions: on-site (four items) and off-site (three survey items) PEBs. The former assessed actions specific to environmentally responsible behavior within the park setting (e.g., “When walking in Namsan Park, I try to minimize environmental damage”), while the latter addressed habitual eco-friendly practices in everyday life (e.g., “I try to reduce water and electricity use at home”). All items were rated using a 5-point Likert-type scale. Additionally, the survey included questions about respondents’ sociodemographic information such as age, gender, income, and education level. All items in our survey were rated on a 5-point Likert-type scale (1 = strongly disagree ~5 = strongly agree). The indicators used to assess validity and reliability, including factor loadings and Cronbach’s alpha coefficients, are presented in Table 1. Considering both the content of the items and the values of these indicators, all measures were found to be at an acceptable level. We assessed internal consistency using Cronbach’s alpha coefficients; all Cronbach’s alpha coefficients were greater than 0.60, which confirmed the internal consistency of the measured variables (Hair et al., 1998) (see Table 1). All items were analyzed as individual indicators of latent constructs in the SEM; no sum or mean scores were created. The descriptive statistics (M, SD) in Table 1 are presented at the item level.

**Table 1:** Means, standard deviations, internal consistencies, and factor loadings for Climate change perception, Place attachment, and Pro-environmental behavior.

Variables/Survey items	SNF				*α*
	*M*(SD)	*λ*	AVE	CR	
*Climate change perception*			0.803	0.942	0.918
a. Climate change will negatively affect my leisure activities (such as walking in Namsan Park).	3.99 (1.05)	0.865			
b. I will make everyday efforts to help prevent climate change.	4.23 (0.69)	0.815			
c. Climate change will lead to negative consequences, such as causing global pandemics.	4.39 (0.77)	0.836			
d. Climate change poses a threat to ecosystems.	4.55 (0.68)	0.694			
*Place identity*			0.802	0.924	0.876
a. Namsan Park means a lot to me.	4.43 (0.77)	0.824			
b. I feel a strong attachment to Namsan Park.	4.40 (0.80)	0.858			
c. When I am in Namsan Park, I feel a sense of self and connection with the place.	4.37 (0.76)	0.724			
*Place dependence*			0.709	0.907	0.863
a. I enjoy coming to Namsan Park more than going to other places.	4.29 (0.81)	0.758			
b. I feel more satisfied when I visit Namsan Park than when I visit other parks.	4.32 (0.77)	0.790			
c. Visiting Namsan Park is more important to me than going to other places.	9.93 (0.95)	0.704			
d. Namsan Park cannot be replaced by other places.	3.76 (1.15)	0.586			
*Social bonding*			0.738	0.849	0.655
a. I have many enjoyable memories with my friends at Namsan Park.	4.27 (0.82)	0.832			
b. I have had special experiences with others in Namsan Park.	4.08 (0.92)	0.644			
*On-site (park-specific) PEB*			0.679	0.894	0.841
a. I will minimize environmental damage while walking in Namsan Park.	4.72 (0.49)	0.710			
b. I will try to prevent the spread of harmful plants and animals in Namsan Park’s ecosystem.	4.49 (0.65)	0.579			
c. I will try not to litter in Namsan Park.	4.79 (0.44)	0.761			
d. I will avoid throwing substances that could pollute Namsan Park.	4.81 (0.44)	0.665			
Off-site (daily life) PEB			0.640	0.842	0.717
a. I usually separate and recycle paper, plastic, and cans.	4.66 (0.54)	0.593			
b. I try to conserve water and electricity at home.	4.32 (0.79)	0.757			
c. I use environmentally friendly products.	3.99 (0.82)	0.571			

Scales ranged from 1 (Strongly Disagree) to 5 (Strongly Agree).

Fit Statistics: *χ*^2^ = 175.112, df = 78, RMSEA = 0.058, NNFI = 0.978, CFI = 0.984.

### 3.4 Analyses

We used confirmatory factor analysis (CFA) to validate the theorized factor structure of our measurement model, and the model fit indices were examined to confirm the variable composition. All analyses were conducted using LISREL 8.70 and R.4.2.1. We evaluated the composite reliability (CR) of all dimensions and the average variance extracted (AVE), aiming for values above 0.70, 0.50, respectively (Fornell and Larcker, 1981). Convergent validity was confirmed as all factor loadings exceeded 0.50, AVE values were above 0.50, and CR values were greater than 0.70. Discriminant validity was also supported, as the square roots of the AVE values for all constructs were greater than their corresponding inter-construct correlations (see Tables 1 and 2), satisfying the Fornell–Larcker criterion (Fornell and Larcker, 1981; Lee et al., 2014).

**Table 2:** The results of correlation analysis.

	1	2	3	4	5	6
1. Climate change perception	1					
2. Place identity	0.250^***^	1				
3. Place dependence	0.175^**^	0.637^***^	1			
4. Social bonding	0.215^***^	0.589^***^	0.546^***^	1		
5. On-site pro-environmental behavior	0.354^***^	0.437^***^	0.397^***^	0.363^***^	1	
6. Off-site pro-environmental behavior	0.268^***^	0.416^***^	0.370^***^	0.331^***^	0.502^***^	1

^**^p < 0.01, ^***^p < 0.001.

We used structural equation modeling (SEM) to test the hypothesized structural relationships. In addition, to test the mediating effect of the two dimensions of place attachment (i.e., place identity and social bonding) on the hypothesized relationship, we used the bootstrap method to measure mediating effects (H3). We set the bootstrap confidence interval (CI) at 95% and the number of bootstrap samples was 5,000. If zero was not included in the interval of 95% CI, it indicated that the mediating effect was significant (MacKinnon, 2008). The hypothesized model was assessed using the following goodness-of-fit indices: root mean square error of approximation (RMSEA under 0.10) (MacCallum et al., 1996), non-normed fit indices (NNFI > 0.90) (Hu and Bentler, 1998), and comparative fit indices (CFI > 0.95) (Hu and Bentler, 1998).

In addition, we examined potential moderation effects of gender and age using a two-stage latent interaction approach. All focal predictors were mean-centered, and product indicators were created for interaction terms (e.g., climate change perception × Gender, climate change perception × Age, place identity × Gender, place identity × Age, social bonding × Gender, social bonding × Age). Structural models including these interactions were estimated, and coefficients were evaluated using robust standard errors and bias-corrected bootstrap confidence intervals (5,000 resamples, 95% CI). Conditional indirect effects were probed to determine whether moderation occurred on the a-paths (e.g., climate change perception → social bonding by Age) or b-paths (e.g., social bonding → on-site/off-site PEB by Age).

## 4 Results

### 4.1 Descriptive analysis

The demographic characteristics of the survey respondents who participated in this study were examined and presented in Table 3. Among the participants, 52% were male and 48% were female. A majority (51.3%) reported having completed a university degree. In terms of occupation, 20.6% of the respondents were homemakers, 20.3% worked in office jobs, and approximately 18.9% were professionals. Regarding annual income, the largest group (18.6%) reported earning between KRW 30,000,000 and 39,999,999 (approximately USD 22,500–30,000), followed by those earning between KRW 50,000,000 and 59,999,999 (approximately USD 37,500–45,000), accounting for 11.1%. Considering the inclusion of homemakers, about 10.3% of respondents reported earning less than KRW 12,500,000 (approximately USD 9,400) per year.

**Table 3:** Socio-demographic characteristics.

Characteristics	Category	Valid percent
Gender	Male	52.0
	Female	48.0
Age	Mean = 56.4 (SD = 14.3)	
Education	Under high school	20.2
	Two-year college	8.9
	University graduate	51.3
	Master’s degree and more	19.6
Occupation	College student	4.1
	Office job	20.3
	Homemaker	20.6
	Self-employment	12.6
	Professional	18.9
	Freelance	6.3
	Unemployed	7.2
	Other	10.0
Annual income	<KRW 10,000,000 (approx. USD 9,400)	10.3
	KRW 10,000,000–19,990,000	3.9
	KRW 20,000,000–29,990,000	5.0
	KRW 30,000,000–39,990,000	18.6
	KRW 40,000,000–49,990,000	8.2
	KRW 50,000,000–59,990,000	11.1
	KRW 60,000,000–69,990,000	8.6
	>KRW 70,000,000	34.3

### 4.2 Correlation between variables

Table 2 presents the Pearson correlation coefficients among the key variables. All correlations were statistically significant and in the expected positive direction. Pearson’s *r* values ranged from 0.175 to 0.637, indicating small to strong associations among climate change perception, dimensions of place attachment, and both on-site and off-site PEBs.

### 4.3 Structural equation model (SEM) testing

Prior to testing the structural model, we assessed the potential threat of common method bias (CMB). Both procedural and statistical remedies were applied (Podsakoff et al., 2003, 2012). In particular, we examined the variance inflation factor (VIF) values following Kock’s (2015) procedure. All VIF values were between 1.64 and 1.97, which are below the recommended threshold of 3.3, suggesting that CMB was not a serious concern in this study (Kang et al., 2021). Based on the confirmatory factor analysis (CFA), all six variables—climate change perception, the three dimensions of place attachment, and the two dimensions of PEB—demonstrated a good model fit with the 20 survey items (*χ*^2^ = 175.112, df = 78, RMSEA = 0.058, NNFI = 0.978, CFI = 0.984). These indices met the recommended fit criteria, confirming the validity of the measurement model. The structural model also showed an acceptable fit (*χ*^2^ = 494.337, df = 155, RMSEA = 0.080, NNFI = 0.899, CFI = 0.917) (Table 4), supporting the hypothesized relationships. First, climate change perception positively influenced place identity (H1; *β* = 0.280, *p* < 0.001), place dependence (H2; *β* = 0.192, *p* < 0.001) and social bonding (H3; *β* = 0.287, *p* < 0.001). Second place identity positively influenced on-site PEB (H4; *β* = 0.223, *p* < 0.05) and off-site PEB (H6; *β* = 0.201, *p* < 0.05). Third, social bonding positively influenced on-site PEB (H5; *β* = 0.223, *p* < 0.05).

**Table 4:** Hypotheses testing.

Hypothesis	*R* ^2^	*β*	S.E.
H1: Climate change perception → Place Identity	0.078	0.280^***^	0.178
H2: Climate change perception → Place dependence	0.037	0.192^***^	0.137
H3: Climate change perception → Social Bonding	0.183	0.287^***^	0.183
H4: Place Identity → On-site Pro-environmental behavior	0.223	0.211^*^	
H5: Social Bonding → On-site Pro-environmental behavior	0.223	0.230^*^	
H6: Place Identity → Off-site Pro-environmental behavior	0.201	0.245^*^	

^*^p < 0.05, ^***^p < 0.001.

We further analyzed the indirect effects to examine whether place attachment was a significant mediator of the relationship between the climate change perception and two types of PEBs. Bootstrapping results provided support for the relationships among the variables (Table 5). The confidence intervals for all indirect effects did not include zero, suggesting that each of the three paths in the hypothesized model showed statistically significant indirect effects. These findings provide empirical evidence that climate change perception positively influenced off-site PEB indirectly through place identity (path 2: indirect effect = 0.150, 95%CI = [0.077, 0.230], *p* < 0.01) and social bonding (path 3: indirect effect = 0.139, 95%CI = [0.058, 0.231], *p* < 0.01).

**Table 5:** Summary of indirect effects of place attachment (place identity, place dependence and social bonding) on the relationship between climate change perception and pro-environmental behaviors using bootstrapped 95% confidence intervals (lower and upper bounds).

Path	Indirect effect	Direct effect	S.E.	95% C.I.	Total effect
CCP → PI → ONPEB	0.142^***^	0.409	0.0378	(0.073, 0.221)	0.551
CCP → PI → OFFPEB	0.150^***^	0.328	0.0390	(0.077, 0.230)	0.478
CCP → SB → OFFPEB	0.139^**^		0.0436	(0.058, 0.231)	0.467

^**^p < 0.01, ^***^p < 0.001.

PI, place identity; SB, social bonding; CCP, climate change perception; ONPEB, on-site pro-environmental behaviors; OFFPEB, off-site pro-environmental behaviors.

In addition, the indirect effects of climate change perception on on-site PEB were positively mediated by place identity (path 1: indirect effect = 0.142, 95%CI = [0.073, 0.221], *p* < 0.001). Since both the direct and indirect effects remained significant, place identity partially mediated the relationships between climate change perception and both on-site and off-site PEBs. Social bonding also served as a partial mediator of the link between climate change perception and off-site PEB, whereas place dependence did not show a significant mediating effect.

### 4.4 Direct effects

Bias-corrected bootstrapping indicated that the climate change perception → off-site PEB was significant (*β* = 0.267, *t* = 4.74, 95% CI [0.167, 0.389]) and the climate change perception → on-site PEB was also significant (*β* = 0.364, *t* = 6.18, 95% CI [0.255, 0.484]). The direct effect was stronger for on-site PEB than for off-site PEB, consistent with the idea that climate-related beliefs translate more forcefully into context-bound stewardship while still generalizing to everyday practices. Together with the significant indirect effects via place identity and social bonding (Section 4.3), these results indicate a partial-mediation pattern.

### 4.5 Gender as a moderator

Using the two-stage latent interaction approach, none of the gender interactions reached significance: climate change perception × Gender **→** place identity (*β* = 0.041, 95% CI [−0.107, 0.222]), place identity × Gender **→** on-site PEB (*β* = −0.054, 95% CI [−0.220, 0.107]), place identity × Gender **→** off-site PEB (*β* = 0.010, 95% CI [−0.129, 0.143]), and social bonding × Gender **→** on-site PEB (*β* = 0.012, 95% CI [−0.104, 0.136]) were all non-significant (all |*t*| < 1; 95% CIs included 0). Consistently, the indirect effects (climate change perception **→** place identity **→** on-site PEB, climate change perception **→** place identity **→** off-site PEB, climate change perception **→** social bonding **→** on-site PEB) did not differ by gender based on bootstrap contrasts. Thus, gender did not moderate the three focal mediation paths in this study.

### 4.6 Age as a moderator

Moderated mediation models incorporating age yielded a largely similar pattern: the baseline mediation effects (climate change perception **→** place identity **→** on-site PEB, climate change perception **→** place identity **→** off-site PEB, climate change perception **→** social bonding **→** on-site PEB) were supported, and most age interaction terms were non-significant. One exception emerged on the a-path to social bonding: climate change perception × Age **→** social bonding showed a negative interaction (*β* = −0.146, *p* < 0.05, 95% CI [−0.275, −0.015]), indicating that the positive effect of climate-change perception on SB weakens with higher age. However, age did not moderate the b-paths (place identity **→** on-site PEB/off-site PEB, social bonding **→** on-site PEB/off-site PEB were non-significant for age interactions), and the conditional indirect effect via SB (climate change perception **→** social bonding **→** on-site PEB/off-site PEB) did not reach significance across observed age values in bootstrap probing. Taken together, age shows a limited moderating influence confined to climate change perception **→** social bonding, and we do not conclude a statistically significant moderated mediation for the SB channel in the present data.

## 5 Discussion

This study provides important empirical insight into how individuals’ perceptions of climate change influence their PEBs through different dimensions of place attachment. In particular, the findings underscore that emotional (place identity), functional (place dependence), and social (social bonding) bonds with an urban park like Namsan serve as meaningful psychological mechanisms in translating climate-related awareness into concrete environmental actions. In addition, supplementary analyses showed significant direct paths from the climate change perception to both on-site PEB and off-site PEB—stronger for on-site PEB—corroborating partial mediation and underscoring the contextual salience of parks for converting climate concern into stewardship. We also explored potential moderators requested by reviewers. Gender did not condition any of the mediation pathways. Age exhibited a small, negative interaction only on the climate change perception and social bonding path, suggesting that older visitors translate climate concern into social-bonding experiences somewhat less strongly; however, no moderated mediation was detected for the downstream indirect effects. These results indicate that the core mediation pattern is broadly robust across gender and age in this sample.

First, the results demonstrate that individuals who perceive climate change as a serious environmental threat are more likely to develop emotional, functional, and social connections to Namsan Park. This suggests that concern about environmental degradation may heighten the personal significance of natural spaces within the city, reinforcing not only symbolic identification with the place but also practical reliance on its restorative and recreational functions. Moreover, climate-aware individuals tend to form stronger interpersonal connections in the park, indicating that shared environmental values and experiences foster social cohesion in public green spaces. Consistent with these findings, Tenbrink and Willcock (2023) report that individuals who perceive environmental threats exhibit stronger attachment to places upon which they depend, suggesting that climate concern enhances both emotional bonding and functional reliance on natural settings. Additionally, empirical evidence (Bazrafshan et al., 2021) indicates that heightened environmental perception is positively correlated with place attachment, reinforcing emotional, functional, and social ties to green spaces.

Second, these place-based attachments contribute to PEB in distinct ways. Emotional identification with Namsan Park—reflected in place identity—was found to influence both on-site (park-specific) behaviors (e.g., minimizing ecological harm while visiting) and off-site (daily life) environmental actions such as recycling or reducing resource use. This dual impact highlights the broad motivational role of place identity, which seems to anchor sustainable behavior not only to a specific setting but also to an individual’s lifestyle. This aligns with empirical findings demonstrating that place identity—a core component of place attachment—directly predicts PEBs, such as park-specific conservation actions and everyday recycling or resource reduction (Chen and Liu, 2024).

Third, social bonding within the park environment plays a critical role in shaping environmentally responsible actions during park visits. When individuals share experiences and environmental values with others, these interactions appear to reinforce a collective sense of responsibility toward space. This aligns with the idea that social norms and group identity can act as enablers of sustainable behavior, particularly in settings where people regularly encounter one another over shared environmental concerns. This is supported by empirical evidence showing that the social-bonding dimension of place attachment in urban parks is particularly effective at promoting pro-environmental behavior—especially higher-effort conservation actions—by reinforcing collective responsibility through shared experiences and group norms (Soopramanien et al., 2023).

Fourth, functional dependence on the park—defined by its ability to fulfill specific needs—contributed to PEB within the park setting. Although its effect did not extend to general behaviors outside the park, this finding reinforces the view that perceived irreplaceability and utility of a place can motivate users to engage in protective behaviors during use. It also reflects the importance of physical access and environmental quality in shaping users’ behavioral commitments. This finding is in line with Wan et al. (2022), who demonstrated that place dependence—reflecting the functional utility and irreplaceability of a setting—significantly predicted site-specific PEB, such as recycling, even when its influence on broader behavioral intentions was limited.

Fifth, the study found that both place identity and social bonding mediated the relationship between climate change perception and PEB. These mediation effects were evident in both everyday and site-specific behavioral domains. This supports the notion that place attachment functions as a psychological bridge that links abstract environmental concerns to tangible, habitual actions (Cheng and Wu, 2015). Particularly in highly urbanized environments, cultivating place-based emotional and social connections may be essential for fostering sustained climate-conscious behavior (Perry et al., 2021; Tenbrink and Willcock, 2023).

Taken together, these findings offer compelling evidence that climate change awareness does not operate in isolation but is activated and amplified through meaningful connections to place. Urban parks like Namsan thus represent more than physical amenities—they serve as psychosocial spaces where environmental attitudes are internalized and enacted. This study not only reaffirms the importance of place attachment in environmental psychology but also expands its relevance to everyday urban leisure settings.

### 5.1 Implications

This study offers several theoretical and practical implications for the fields of environmental psychology, leisure studies, and urban sustainability planning. The findings advance the theoretical understanding of how climate change perception influences PEB through the mediating role of place attachment. While prior research has highlighted the direct influence of environmental attitudes on behavior, this study demonstrates that emotional (place identity), functional (place dependence), and social (social bonding) connections to place serve as distinct and significant pathways linking climate concern to behavioral outcomes. In particular, the simultaneous examination of both on-site (park-specific) and off-site (daily life) behaviors provides a more nuanced model of environmental engagement, extending the conceptual boundaries of place attachment theory. Moreover, by confirming the mediating role of place identity and social bonding, the study highlights the importance of affective mechanisms in translating cognitive environmental concern into sustained action.

From a policy and planning perspective, the results underscore the critical role of urban parks like Namsan as catalysts for fostering environmental stewardship. Given that place attachment enhances both park-specific and general PEB, park managers and urban designers should actively cultivate opportunities for emotional and social engagement within park spaces. Strategies may include developing educational signage about climate change, supporting community-based environmental programs, and designing inclusive spaces that facilitate shared experiences among users. Additionally, communication campaigns aimed at highlighting the ecological and personal value of urban green spaces can strengthen residents’ place identity and encourage eco-friendly practices beyond the park. Ultimately, these findings suggest that leveraging psychological bonds to place can serve as a cost-effective and scalable approach to encouraging sustainable behavior in urban populations.

### 5.2 Limitations and future research

This study examined the role of restorative perceptions and policy support in the context of fine dust risk, while incorporating emotional factors such as place attachment and PEB. However, several limitations must be acknowledged. First, the study was conducted in a specific urban park—Namsan Park in Seoul. While this site offers valuable insights into urban park dynamics, visitor responses may differ in other types of natural settings, such as mountainous or semi-wild environments. For example, Bukhansan National Park in South Korea, located on the urban fringe, offers deeper nature immersion, which may enhance place bonding and strengthen pro-environmental behavioral intentions (Korpela et al., 2008). Future research should compare diverse environmental contexts, including urban forests like Seoul Forest and more remote natural areas, to explore how setting characteristics shape psychological and behavioral outcomes. Second, the structured survey method limited exploration of visitors’ emotional narratives or behavioral motivations. Employing qualitative or mixed-method designs—such as in-depth interviews, photo-elicitation, or ethnographic walking methods—could yield richer insights into how restorative experiences translate into sustained environmental action. Third, the study used a cross-sectional design, which restricts causal interpretation. To observe how restorative experiences, place attachment, and PEB develop over time, longitudinal or experimental approaches are recommended (Hartig et al., 2014). Such methods could help clarify whether and how repeated exposure to restorative environments reinforces environmental identity and long-term civic engagement. Fourth, the participant sample was relatively narrow—mainly adult walkers in an urban setting. Future studies should include a broader demographic spectrum, capturing variations across age groups and cultural backgrounds. For instance, children, older adults, or tourists may exhibit distinct environmental perceptions or weaker place attachment, influencing their behavioral outcomes differently. By addressing these limitations, future research can more comprehensively integrate the emotional, spatial, and behavioral dynamics of environmental engagement, ultimately contributing to evidence-based green space design, urban resilience, and climate-responsive planning.

## 6 Conclusion

This study demonstrated that urban park visitors’ climate change perception significantly influences both their daily and site-specific PEBs, and that this relationship is mediated by emotional and functional dimensions of place attachment—namely, place identity, place dependence, and social bonding. By integrating climate change perception with the concept of place attachment, the study highlights the importance of emotional and spatial bonds in promoting sustainable behaviors in leisure settings. Unlike previous studies that often examined either emotional or functional attachment in isolation, this study integrates multiple dimensions of place attachment to provide a more holistic understanding of how urban leisure settings can foster climate-conscious behaviors. The findings emphasize that fostering strong psychological connections to urban green spaces can be an effective strategy to enhance environmental stewardship. These insights offer valuable implications for urban park planning and environmental policy, suggesting that interventions aimed at strengthening place attachment may serve as a meaningful pathway to increase public engagement in pro-environmental actions amidst escalating climate risks.

## Data Availability

The raw data supporting the conclusions of this article will be made available by the authors, without undue reservation.
